# Synthesis, docking study and biological evaluation of some new thiourea derivatives bearing benzenesulfonamide moiety

**DOI:** 10.1186/s13065-017-0271-7

**Published:** 2017-05-19

**Authors:** Mostafa M. Ghorab, Mohamed S. A. El-Gaby, Aiten M. Soliman, Mansour S. Alsaid, Marwa M. Abdel-Aziz, Mahmoud M. Elaasser

**Affiliations:** 1 Department of Pharmacognosy, College of Pharmacy, King Saud University, P.O. Box 2457, Riyadh, 11451 Saudi Arabia; 2 Department of Drug Radiation Research, National Center for Radiation Research and Technology, Atomic Energy Authority, Cairo, 113701 Egypt; 3 Department of Chemistry, Faculty of Science, Al-Azhar University, Assiut, 71524 Egypt; 4 The Regional Center for Mycology and Biotechnology, Al-Azhar University, Cairo, Egypt

**Keywords:** Thiourea, Sulfonamides, Structure–activity relationship, Antimycobacterial

## Abstract

**Background:**

A series of novel *N*-(2, 6-dimethoxypyrimidin-4-yl)-4-(3-(aryl)thioureido) benzenesulfonamides **3a**–**t** was synthesized by the addition of *N*-(2,6-dimethoxypyrimidin-4-yl)-4-isothiocyanatobenzenesulfonamide **2** to the appropriate aromatic amine. The structures of the synthesized compounds were inspired from the second line antituberculosis pro-drugs.

**Results:**

Most of the new compounds were screened for their activity against *Mycobacterium tuberculosis*. The results of the antimycobacterial assay showed that compound **3i** exerted the highest activity (MIC = 3.13 µg/mL), followed by compound **3s** (MIC = 6.25 µg/mL).

**Conclusion:**

The structure–activity relationship (SAR) analysis revealed that the introduction of the benzo[1,3]dioxol moiety in **3i** and the 4-morpholinyl-4-phenyl moiety in **3s** has proven to give the most potent compounds in this study. Docking of the promising compounds inside the active site of *M. tuberculosis* enoyl reductase InhA was performed in order to emphasize the results. The compounds showed a similar orientation to that of GSK 625 inside the active site of **5JFO** and bind to Met 98 in a way similar to that of the co-crystallized ligand.

## Background

Tuberculosis (TB), is a disease caused by the facultative intracellular bacterium called *Mycobacterium tuberculosis* (MTB). WHO declared TB as a global health crisis [1] and a main cause of death due to the lack of appropriate treatment against resistant strains [2]. In 2012, TB was responsible for the death of 1.3 million people worldwide, Over 95% of them were from developing countries, also, TB represents the third cause of death for women aged 15–44. In addition, about one-third of the world’s population harbors a dormant MTB infection, representing a significant incidence of the disease for the future [3]. TB treatment is tedious and time-consuming, that requires direct therapy and follow-up for not less than 6 months using these four drugs (isoniazid, rifampicin, pyrazinamide and ethambutol [1, 4]. In addition, the recurrences of latent tuberculosis, are particularly prevalent in individuals with compromised immune system [5]. However, the present treatment protocols have proven to be underwhelming due to drug–drug interactions, intolerance, drug toxicity and poor patient adherence due to the lengthy treatment protocols [1, 6]. That’s why more effective and shorter treatment regimens are required.

**Fig. 1:**
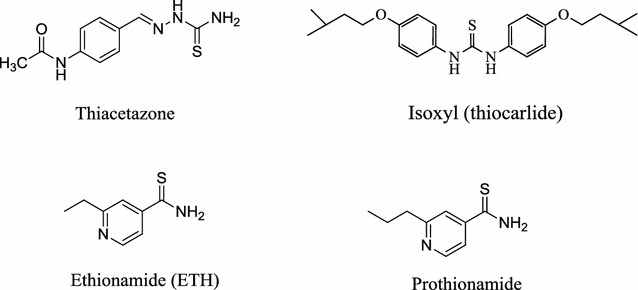
Second line antituberculosis pro-drugs

Thioureas act as precursors for the synthesis of different classes of acyclic and heterocyclic compounds, in addition to their high biological activity [7–10]. Second line antituberculosis pro-drugs as thioacetazone which is useful in preventing resistance to more powerful drugs such as isoniazid, isoxyl (thiocarlide) that is effective against multi-drug resistant strains, ethionamide (ETH) and prothionamide (Fig. 1) [11–17], were used to inspire the structures of our new thiourea derivatives, together with their mode of action. On the other hand, sulfonamides were largely employed as preventive and chemotherapeutic agents against various diseases [18], recent studies have shown that sulfonamides also possess antimycobacterial activity [19].

For the above-mentioned reasons and as a part of our interest in the synthesis and screening of potentially bioactive compounds [20–24], we herein, report the synthesis of some novel *N*-(2,6-dimethoxypyrimidin-4-yl)-4-(3-(aryl)thioureido)benzenesulfonamides **3a**–**t** to be evaluated for their antimycobacterial activity. The promising compounds **3i** and **3s** were docked inside the active site of *M. tuberculosis* enoyl reductase InhA, to predict their possible mode of action. InhA enzyme was chosen as it contains a very hydrophobic site that favorably interacts with thioamide or thiourea moieties [25].

## Results and discussion

### Chemistry

**Scheme 1:**
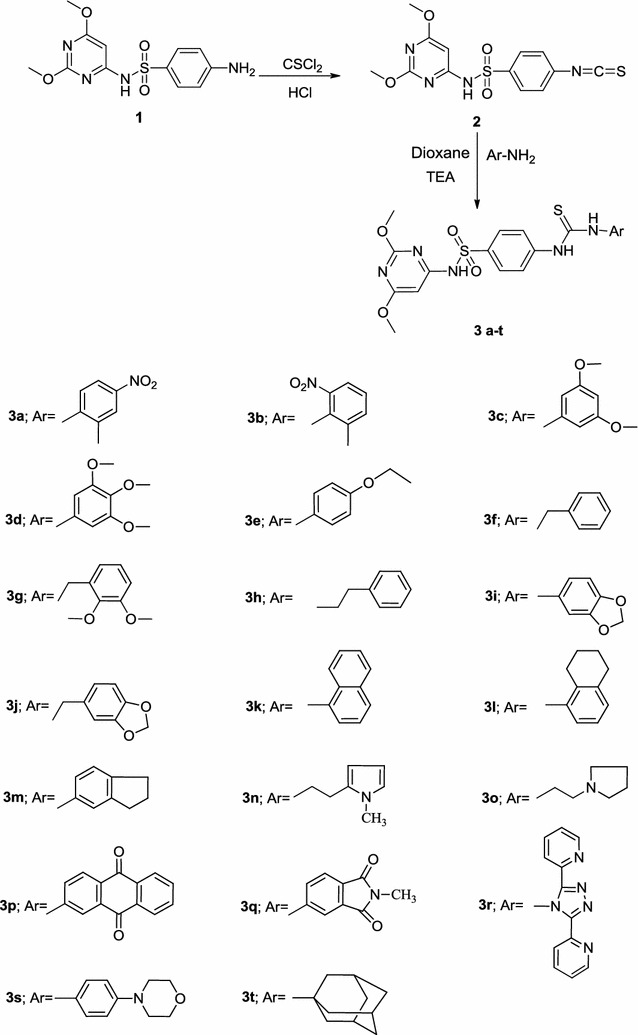
Synthesis of the thiourea derivatives **3a**–**t**

Isothiocyanates are widely used building blocks in the synthesis of nitrogen, sulfur and oxygen heterocycles [26]. The high electrophilicity and nucleophilicity associated with the carbon and sulfur atoms, respectively, of the isothiocyanates and their extended π electron system make them unique precursors for a large variety of target molecules. The intermediate, *N*-(2,6-dimethoxypyrimidin-4-yl)-4-isothiocyanatobenzenesulfonamide **2** [27] used for the preparation of the target compounds have been synthesized via thiophosgenation of sulfadimethoxine **1** at room temperature in the presence of dilute hydrochloric acid, according to the reported procedure (Scheme 1).

A series of *N*-(2,6-dimethoxypyrimidin-4-yl)-4-(3-(aryl)thioureido) benzenesulfonamides **3a**–**t** was prepared by condensation of aromatic amines with *N*-(2,6-dimethoxypyrimidin-4-yl)-4-isothiocyanatobenzenesulfonamide **2** [27] in dioxane at reflux temperature in the presence of catalytic amounts of triethylamine, (Scheme 1). The structures of synthesized compounds **3a**–**t** were confirmed by the absence of characteristic absorption band at 2000–2200/cm (N=C=S). Also, the IR of **3** is characterized by the presence of NH, thiocarbonyl (C=S) and SO_2_ absorption bands. For example, the ^1^H NMR spectrum of compound **3b** showed two singlets at *δ* 3.81 and 3.84 ppm which were assigned for the two methoxy protons, a singlet at *δ* 6.1 ppm assigned to the pyrimidine-H, two downfield shifted singlets at *δ* 11.5 and 11.9 ppm which were readily assigned to the HN(1) and HN(2) protons, in addition to the presence of methyl, SO_2_NH and aromatic protons. The thiocarbonyl group of the thiourea moiety was also observed in the ^13^C-NMR spectrum. The formation of thioureas **3a**–**t** can be explained through the previously reported mechanism [24].

### In vitro antimycobacterial activity evaluation

Evaluation of the synthesized compounds against *M. tuberculosis* (RCMB 010126) was initially carried out using the microplate Alamar blue assay (MABA) at the Regional Center for Mycology and Biotechnology (RCMB), Al-Azhar University (Cairo, Egypt) at a concentration of 200 µg/mL (Table 1). As seen in Table 1, compound **3i** was the most potent analog exhibiting good antimycobacterial activity that produced growth inhibition of 74.9%.

**Table 1:** The inhibitory activities of the synthesized compounds against *Mycobacterium tuberculosis*

Sample code	% Inhibition	SD
**3a**	25.3	1.1
**3b**	0	0
**3c**	0	0
**3d**	8.9	0.3
**3e**	36.2	2.1
**3f**	11.3	0.8
**3g**	14.7	0.6
**3i**	74.9	4.3
**3l**	12.5	1.1
**3n**	0	0
**3o**	0	0
**3p**	23.9	1.4
**3q**	41.2	2.8
**3r**	53.8	2.6
**3s**	59.2	4.3
**3t**	10.3	0.8
Isoniazid	93.5	1.4

The results of the antimycobacterial activity as minimum inhibitory concentration (MIC) are presented in Table 2 and confirming that compound **3i** exerted the highest antimycobacterial activity (MIC = 3.13 µg/mL), followed by compound **3s** (MIC = 6.25 µg/mL) then compounds **3r, 3q, 3e, 3a, 3p, 3g, 3l, 3f, 3t** and **3d**, respectively. On the other hand, compounds **3b**, **3c**, **3n** and **3o** exhibited no antimycobacterial activity under these experimental conditions.

**Table 2:** The estimated minimum inhibitory concentrations (MICs) of the synthesized compounds against *Mycobacterium tuberculosis*

Tested compounds	MIC values (µg/mL)	MIC (µM)
**3a**	50	98.8
**3b**	NA	NA
**3c**	NA	NA
**3d**	200	373.8
**3e**	50	102.2
**3f**	200	435.7
**3g**	100	192.7
**3i**	3.13	6.4
**3l**	200	400.8
**3n**	NA	NA
**3o**	NA	NA
**3p**	100	173.9
**3q**	25	43.5
**3r**	12.5	21.7
**3s**	6.25	11.8
**3t**	200	397.6
Isoniazid	0.195	1.42

*NA* no anti-TB activity under the screening conditions

From the results in Table 2, it is apparent that the 4-position of the thiourea derivatives **3a**–**t**, crucially affected the antimycobacterial activity. In which, incorporation of a Benzo[1,3]dioxol group in compound **3i** led to good activity against *M. tuberculosis* (MIC = 3.13 µg/mL). The introduction of a methoxy group at 2-position of the spirodecane system increased the activity (except for **3b**). The introduction of an electron-donating group at the 4-position, as methyl and methoxy groups, increased the activity. However, di- and trimethoxy substitutions (compounds **3c, 3d** and **3g**) led to decrease in the lipophilicity with a subsequent decrease in the antimycobacterial activity, indicating that the increased lipophilicity is crucial for the antitubercular activity.

It is well documented that increasing the lipophilicity, increases the diffusion through the lipid domain, thus, increasing the efficacy of the antimycobacterial agent [28–31].

### Molecular docking

Tuberculosis is characterized by a number of drug targets namely: InhA, RpoB, DNA Gyrase, ATP synthase, and DprE1, inhibitors of those targets were found to be promising leads [32]. Isoniazid is still the most potent treatment targeting InhA enzyme. Isoniazid was found to interfere with Nicotinamide adenine dinucleotide (NAD)-utilizing enzymes, primarily the enoyl-ACP reductase encoded by the *InhA* gene, leading to the arrest of mycolic acid synthesis, which is essential to *M. tuberculosis* [32, 33]. InhA enzyme was chosen based upon its hydrophobic properties that favorably interact with thioamide or thiourea moieties [25].

**Fig. 2:**
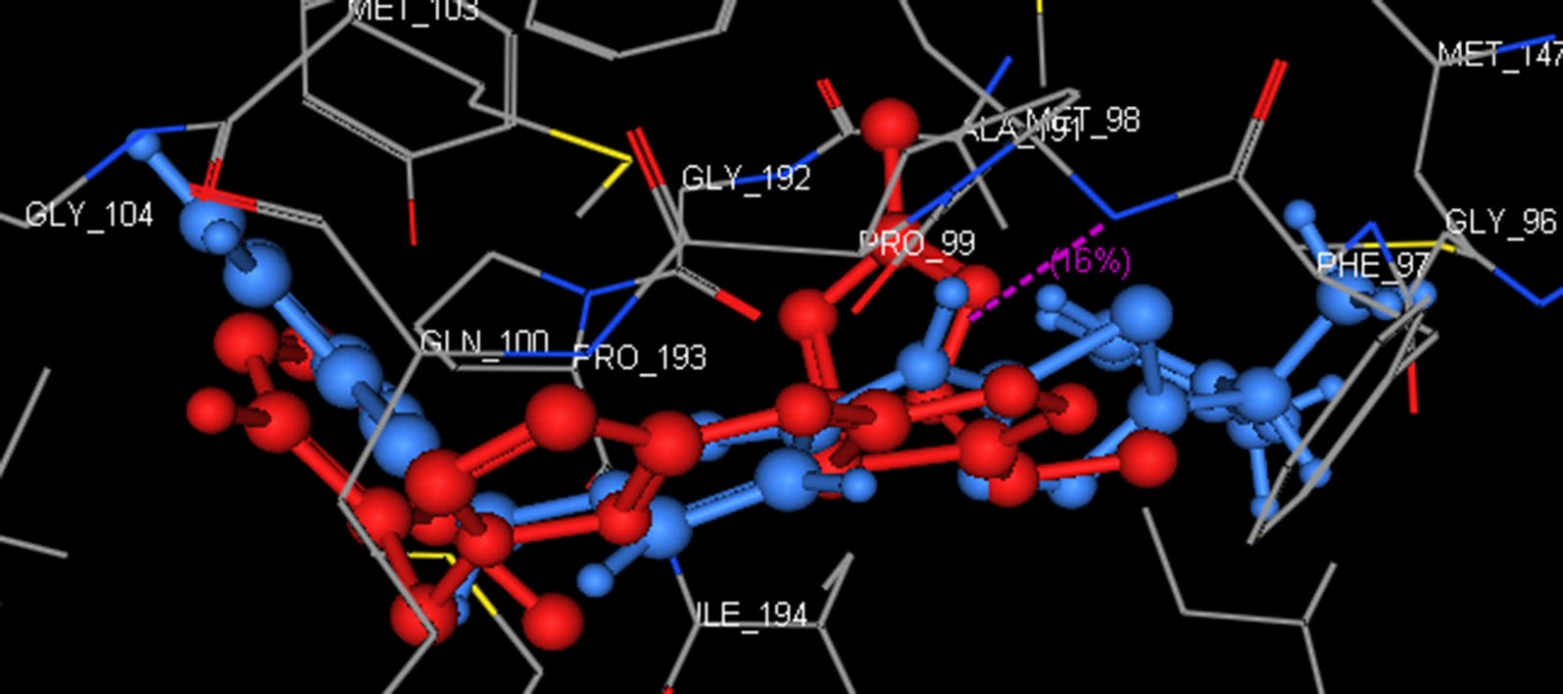
Superimposition of the co-crystallized ligand (*red*) and the re-docked ligand (*blue*) inside the active site of **5JFO**

**Fig. 3:**
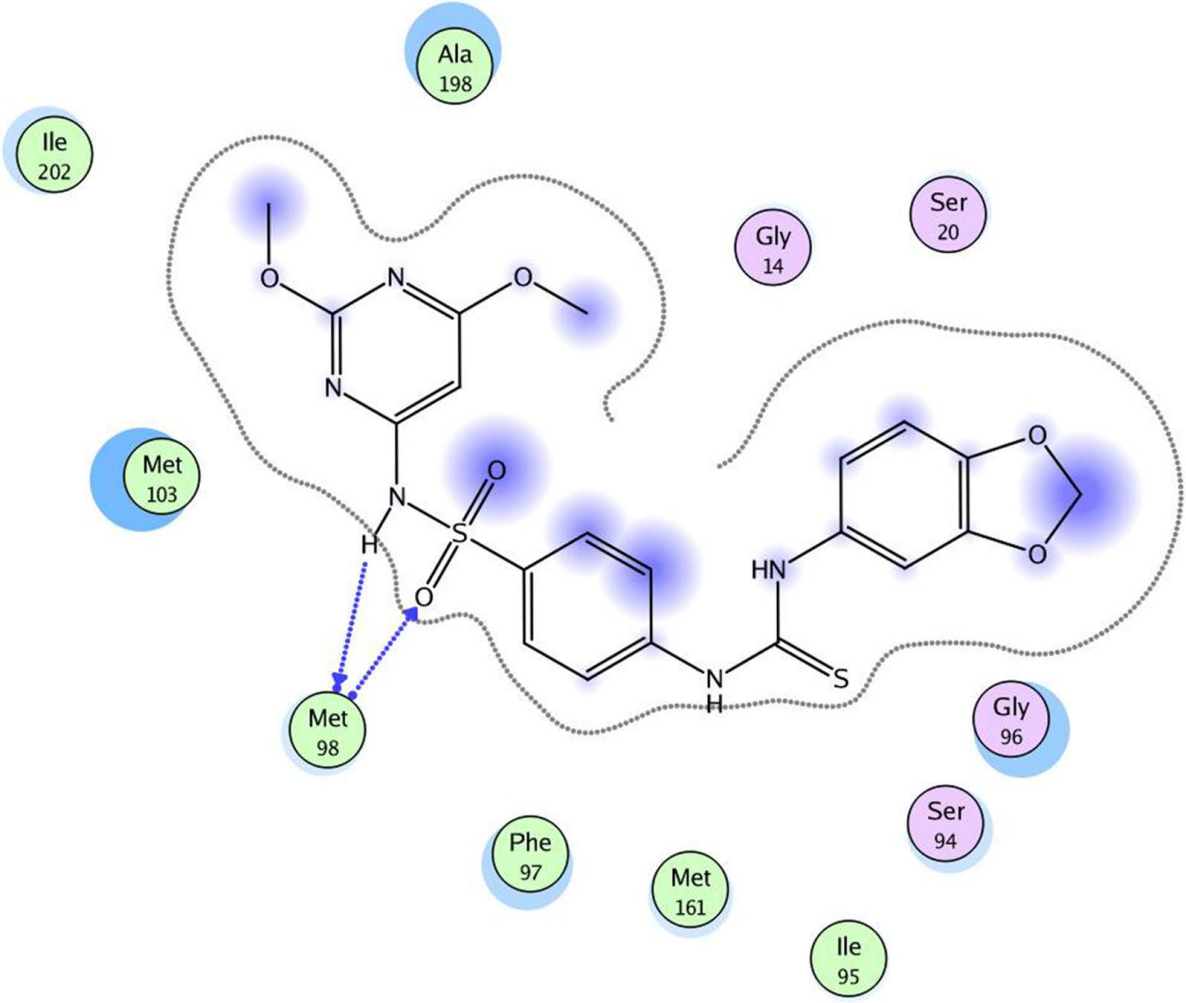
2D interactions of compound **3i** with the active site amino acids of **5JFO**

**Fig. 4:**
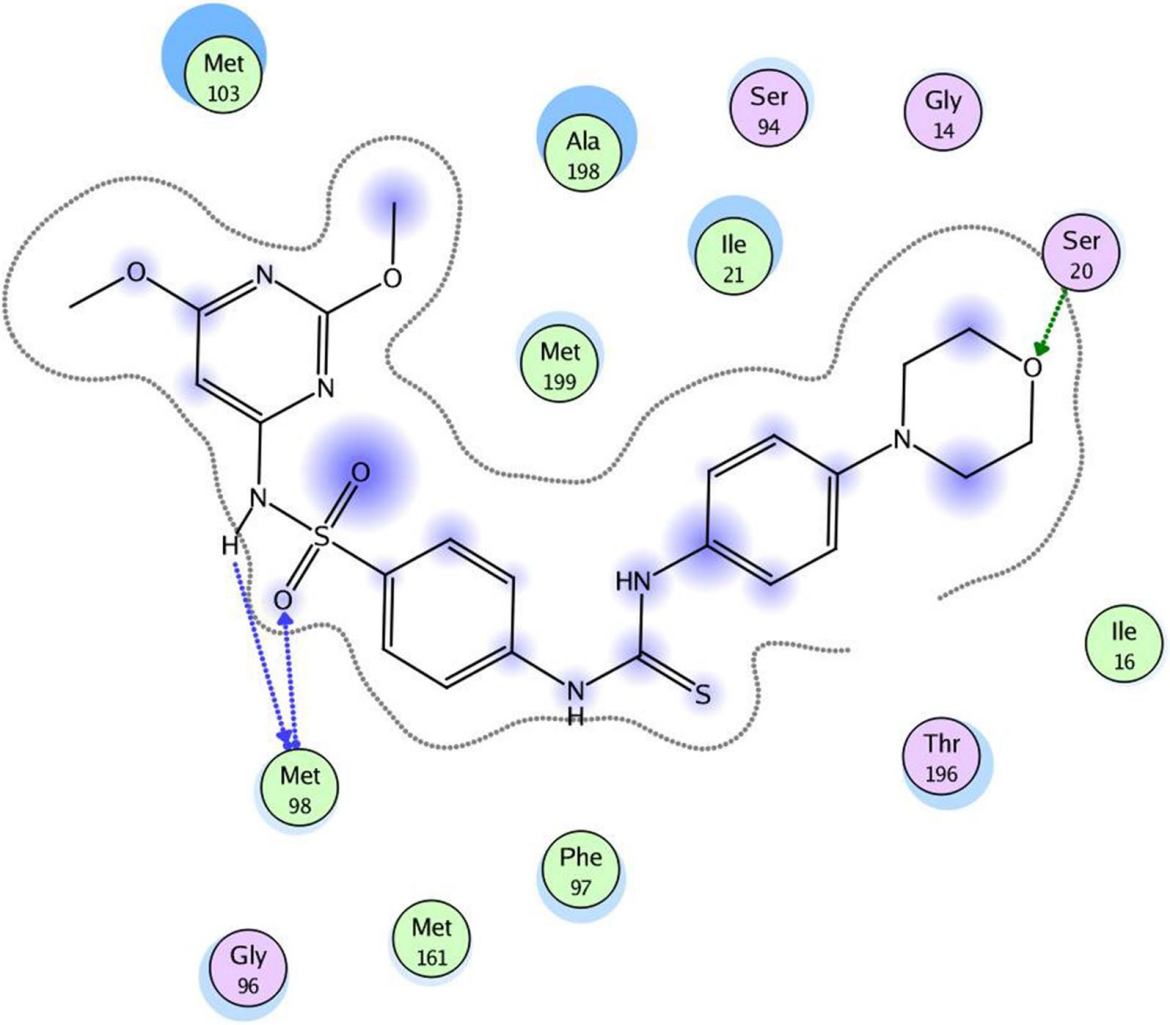
2D interactions of compound **3s** with the active site amino acids of **5JFO**

**Fig. 5:**
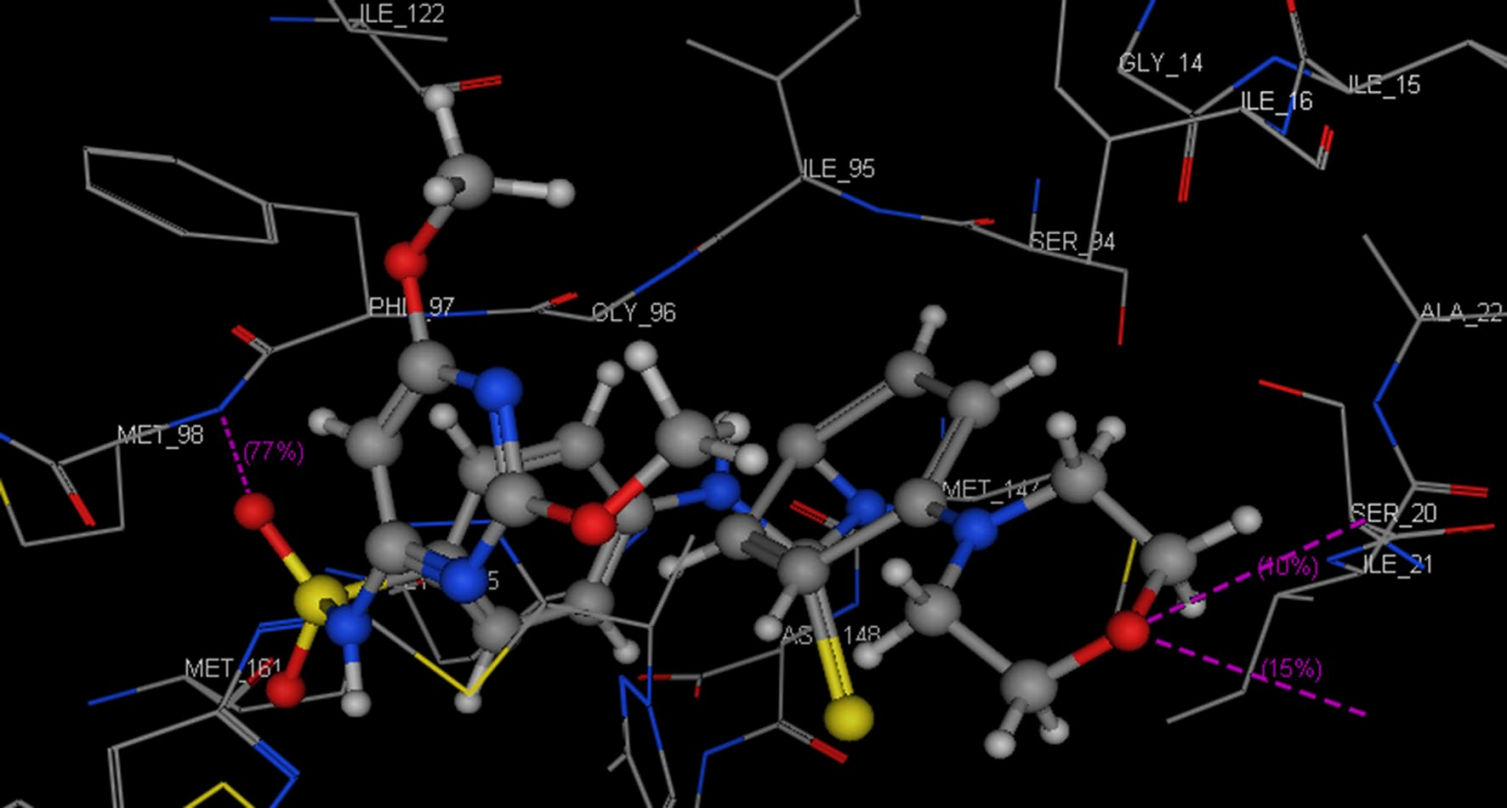
3D docking of compound **3s** (S = −11.64 kcal/mol) in the active site of **5JFO**

In our present study to determine the possible mode of action of the target compounds, molecular docking of compounds **3i** and **3s** was performed in the active site of *Mycobacterium tuberculosis* enoyl reductase InhA to explore their possible binding modes. The protein data bank file (PDB: **5JFO**) was selected for this purpose. The file contains *M. tuberculosis* enoyl reductase InhA enzyme co-crystallized with *N*-[1-[(2-chloro-6-fluorophenyl)methyl]-*1H*-pyrazol-3-yl]-5-[(1S)-1-(3-methyl-*1H*-pyrazol- 1-yl)ethyl]-1,3,4-thiadiazol-2-amine (GSK 625) [34]. All docking procedures were carried out using molecular operating environment (MOE) software 10.2008. Docking protocol was verified by re-docking of the co-crystallized ligand in the binding pocket of the enzyme with energy score (S) = −10.44 kcal/mol and root mean standard deviation (RMSD) = 0.39 (Fig. 2). The 2D ligand interaction of compound **3i** (Fig. 3) demonstrates that the compound binds to the amino acid of the active site Met 98 through two hydrogen bonds (1.72, 2.44 Å). Regarding compound **3s**, the 2D and 3D ligand interaction simulations (Figs. 4 and 5) showed that **3s** binds in the same fashion to the co-crystallized ligand displaying two hydrogen bonds with the active pocket amino acid Met 98 leading to an overall binding energy of = −11.64 kcal/mol (Table 3).

**Table 3:** Docking results of the targeted compounds inside **5JFO** active site

Compound	Energy score (S) (Kcal/mol)	Amino acids	Interacting groups	Length (Å)
Ligand	−10.44	Met 98	C=N	2.72
		Met 98	NH	2.75
**3a**	−8.44	Met 98	SO_2_	3.01
		Met 98	NH	2.14
**3b**	−8.30	Met 98	SO_2_	2.17
		Met 98	NH	3.12
**3c**	−7.89	Met 98	SO_2_	2.63
		Met 98	NH	2.35
**3d**	−9.02	Met 98	SO_2_	1.78
		Met 98	NH	2.54
**3e**	−8.80	Met 98	SO_2_	1.90
		Met 98	NH	2.31
		Ser 19	CO	2.76
**3f**	−8.32	Met 98	SO_2_	3.13
		Met 98	NH	2.85
**3g**	−7.92	Met 98	SO_2_	2.12
		Met 98	NH	2.90
**3i**	−9.07	Met 98	SO_2_	1.72
		Met 98	NH	2.44
**3l**	−7.91	Met 98	SO_2_	2.41
		Met 98	NH	3.04
**3n**	−9.00	Met 98	SO_2_	2.31
		Met 98	NH	2.82
		Thr 17	N–CH_3_	3.08
**3o**	−8.42	Met 98	SO_2_	2.89
		Met 98	NH	3.10
		Ser 20	CO	1.99
**3p**	−8.98	Met 98	SO_2_	2.67
		Met 98	NH	2.71
		Ser 19	CO	3.05
**3r**	−8.14	Met 98	SO_2_	1.83
		Met 98	NH	2.98
		Ser 19	N (triazole)	3.02
		Thr 17	N (triazole)	2.56
**3s**	−11.64	Met 98	SO_2_	2.15
		Met 98	NH	2.65
		Ser 20	CO	3.10
**3t**	−7.88	Met 98	SO_2_	2.73
		Met 98	NH	2.84

### SAR (structure activity relationship)

From the results revealed by the antimycobacterial activity and the docking study, it is apparent that the group attached to the thiourea is crucial for the activity. The benzo[1,3]dioxol derivative **3i** (MIC = 6.4 µM) was the most potent, followed by the 4-morpholinyl-4-phenyl derivative **3s** (MIC = 11.8 µM), the oxygen atom of morpholine binds to Ser 20 inside the active site. Also, **3i** and **3s** have shown similar binding to that of the co-crystallized ligand inside the active site of *M. tuberculosis* enoyl reductase InhA and the best binding score in this series. The dipyridinyl-[1,2,4]triazole **3r** and the 2-methyl-1,3-dioxo-2,3-dihydro-1H-isoindole derivative **3q** also showed potent activity, with MIC = 21.7 and 43.5 µM, respectively. It is apparent that the nitrogens of the triazole ring in **3r** tend to make additional binding interactions inside the active site of the enzyme as well as the carbonyl group in **3q**, which may contribute to their antimycobacterial activity.

## Experimental

### Chemistry

All analyses were performed at King Saud University Research Center (Riyadh, Saudi Arabia). Melting points were determined in open capillaries on a Gallenkamp melting point apparatus (Sanyo Gallenkamp, Southborough, UK). Precoated silica gel plates (Kieselgel 0.25 mm, 60 F254, Merck, Darmstadt, Germany) were used for thin layer chromatography using a developing solvent system of 4:1 chloroform/methanol and the spots were detected by the ultraviolet lamp. IR spectra (KBr discs) were recorded using an FT-IR spectrometer (Perkin Elmer, Waltham, MA, USA). ^1^H-NMR spectra were scanned on NMR spectrometer (Bruker AXS Inc., Flawil, Switzerland), operating at 500 MHz for ^1^H and 125.76 MHz for ^13^C. Chemical shifts are expressed in δ values (ppm) relative to TMS as an internal standard, using DMSO-d6 as a solvent. Mass spectra were recorded on a 600 GC/MS (Clarus, Middletown, CT, USA) and TQ 320 GC/MS/MS mass spectrometers (Varian, West Sussex, UK). Elemental analyses were done on a model 2400 CHNSO analyzer (Perkin Elmer, Waltham, MA, USA). All reagents used were of the analytical grade.

#### General method for the synthesis N-(2,6-dimethoxypyrimidin-4-yl)-4-(3-(aryl)thioureido) benzenesulfonamides **3a**–**t**

A mixture of isothiocyanatobenzenesulfonamide **2** [27] (0.01 mol) with a heterocyclic amine (0.01 mol) was refluxed in dioxane (30 mL) containing triethylamine (0.1 mL) for 1 h. The solvent was evaporated, the solid obtained was washed with petroleum ether (bp 40–60 °C) and crystallized from ethanol to afford the thiourea derivatives.

##### N-(2,6-Dimethoxy-pyrimidin-4-yl)-4-(3-(2-methyl-4-nitro-phenyl)thioureido)benzenesulfonamide (**3a**)

This compound was obtained as yellow powder from ethanol; yield 84%; m.p. 137.9 °C. IR: 3471, 3363, 3230 (NH), 3100 (arom.), 2983, 2817 (aliph.), 1633 (CN), 1396, 1151 (SO_2_), 1290 (CS). ^1^H-NMR: δ 2.1 [s, 3H, CH_3_], 3.79, 3.84 [2s, 6H, 2OCH_3_], 6.5 [s, 1H, H-pyrimidine], 6.8–7.9 [m, 7H, Ar–H], 9.8 [s, 1H, SO_2_NH], 11.4 [s, 2H, 2NH]; ^13^C-NMR: 17.4, 55.4 (2), 80.6, 112.7, 120.7, 124.5 (2), 126.6, 128.4 (2), 133.0, 139.8, 141.3, 143.8 (2), 154.4, 162.6, 169.0, 178.5. Anal. Calcd. for C_20_H_22_N_6_O_6_S_2_: C, 47.42%; H, 4.38%; N, 16.59%; S, 12.66%. Found: C, 47.40%; H, 4.30%; N, 16.50%; S, 12.60%.

##### N-(2,6-Dimethoxy-pyrimidin-4-yl)-4-(3-(2-methyl-6-nitro-phenyl)thioureido) benzenesulfonamide (**3b**)

This compound was obtained as yellow powder from ethanol; yield 82%; m.p. 199.3 °C. IR: 3458, 3371 (NH), 3100 (arom.), 2970, 2831 (aliph.), 1622 (CN), 1392, 1130 (SO_2_), 1251 (CS). ^1^H-NMR: δ 2.1 [s, 3H, CH_3_], 3.81, 3.84 [2s, 6H, 2OCH_3_], 6.1 [s, 1H, H-pyrimidine], 6.8–8.0 [m, 7H, Ar–H], 8.8 [s, 1H, SO_2_NH], 11.5, 11.9 [2s, 2H, 2NH]. ^13^C-NMR: 18.3, 53.4, 53.6, 83.6, 121.7, 123.8 (2), 126.2 (2), 131.2 (2), 136.5(2), 136.9, 141.2, 144.8, 155.6, 167.1, 172.8, 186.3. Anal. Calcd. for C_20_H_22_N_6_O_6_S_2_: C, 47.42%; H, 4.38%; N, 16.59%; S, 12.66%. Found: C, 47.40%; H, 4.30%; N, 16.50%; S, 12.60%.

##### 4-(3-(3,5-Dimethoxyphenyl)thioureido)-N-(2,6-dimethoxy-pyrimidin-4-yl)benzenesulfonamide (**3c**)

This compound was obtained as yellow powder from ethanol; yield 80%; m.p. 179.6 °C. IR: 3437, 3210 (NH), 3100 (arom.), 2920, 2848 (aliph.), 1622 (CN), 1354, 1153 (SO_2_), 1274 (CS). ^1^H-NMR: δ 3.81, 3.82 [2s, 6H, 2OCH_3_, pyrimidine], 3.84, 3.88 [2s, 6H, 2OCH_3_], 5.6 [2s, 3H, CH, dimethoxyphenyl], 6.2 [s, 1H, H-pyrimidine], 6.9–8.1 [m, 4H, Ar–H], 8.9 [s, 1H, SO_2_NH], 9.7 [s, 2H, 2NH]; ^13^C-NMR: 55.1, 55.4, 55.5 (2), 79.4, 98.7, 112.7 (2), 128.1 (2), 131.7 (2), 134.6, 140.7, 142.3, 158.7 (2), 159.1, 163.8, 174.6, 192.4. Anal. Calcd. For C_21_H_23_N_5_O_6_S_2_:C, 49.89%; H, 4.59%; N, 13.85%; S, 12.68%. Found: C, 49.80%; H, 4.50%; N, 13.80%; S, 12.60%.

##### N-(2,6-Dimethoxy-pyrimidin-4-yl)-4-(3-(3,4,5-trimethoxyphenyl)thioureido) benzenesulfonamide (**3d**)

This compound was obtained as Brown powder from ethanol; yield 87%; m.p. 275.5 °C. IR: 3433, 3356 (NH), 3053 (arom.), 2939, 2835 (aliph.), 1616 (CN), 1354, 1128 (SO_2_), 1276 (CS). ^1^H-NMR: δ 3.61, 3.67 [2s, 6H, 2O CH_3_], 3.81, 3.84 [2s, 9H, 3OCH_3_], 5.9 [s, 2H, Ar–H], 6.4 [s, 1H, H-pyrimidine], 6.9–8.3 [m, 4H, Ar–H], 9.7 [s, 1H, SO_2_NH], 11.8 [s, 2H, 2NH]. ^13^C-NMR: 55.2, 55.6, 55.9 (2), 63.7, 83.5, 98.0 (2), 121.6 (2), 128.3 (2), 132.1, 133.7, 135.2, 140.6, 156.0 (2), 158.8, 166.4, 170.3, 181.1. Anal. Calcd. for C_22_H_25_N_5_O_7_S_2_: C, 49.34%; H, 4.70%; N, 13.08%; S, 11.97%. Found: C, 49.34%; H, 4.70%; N, 13.08%; S, 11.97%.

##### N-(2,6-Dimethoxy-pyrimidin-4-yl)-4-(3-(4-ethoxyphenyl)thioureido) benzenesulfonamide (**3e**)

This compound was obtained as Grey powder from ethanol; yield 83%; m.p. 234.2 °C. IR: 3296, 3217 (NH), 3100 (arom.), 2978, 2929, 2873 (aliph.), 1639 (CN), 1390, 1168 (SO_2_), 1246 (CS). ^1^H-NMR: δ 1.2 [t, 3H, CH_3_, *J* = 8 Hz], 3.90, 3.92 [2s, 6H, 2OCH_3_], 4.0 [q, 2H, CH_2_], 6.8 [s, 1H, H-pyrimidine], 7.0–8.4 [m, 8H, Ar–H], 9.5 [s, 1H, SO_2_NH], 11.7 [s, 2H, 2NH]. ^13^C-NMR: 15.2, 53.2, 53.9, 63.5, 84.6, 115.0 (2), 120.3 (2), 127.9 (2), 128.3 (2), 133.3 (2), 142.1, 153.4, 154.0, 164.8, 172.6, 179.3. Anal. Calcd. for C_21_H_23_N_5_O_5_S_2_: C, 51.52%; H, 4.74%; N, 14.30%; S, 13.10%. Found: C, 51.50%; H, 4.70%; N, 14.30%; S, 13.10%.

##### 4-(3-Benzyl-thioureido)-N-(2,6-dimethoxy-pyrimidin-4-yl)benzenesulfonamide (**3f**)

This compound was obtained as yellow powder from ethanol; yield 88%; m.p. > 360 °C. IR: 3365, 3188 (NH), 3034 (arom.), 2981, 2827 (aliph.), 1622 (CN), 1390, 1128 (SO_2_), 1251 (CS). ^1^H-NMR: δ 3.61, 3.64 [2s, 6H, 2OCH_3_], 4.3 [s, 2H, CH_2_], 6.4 [s, 1H, CH pyrimidine], 7.0–8.5 [m, 9H, Ar–H], 9.9 [s, 1H, SO_2_NH], 10.8, 11.7 [2s, 2H, 2NH]. ^13^C-NMR: 49.2, 53.4, 53.8, 85.6, 120.8 (2), 125.7, 127.3 (2), 127.7 (2), 128.8 (2), 139.4 (2), 139.6, 161.5, 167.9, 171.9, 178.8. Anal. Calcd. for C_20_H_21_N_5_O_4_S_2_: C, 52.27%; H, 4.61%; N, 15.24%; S, 13.95%. Found: C, 52.20%; H, 4.60%; N, 15.20%; S, 13.90%.

##### 4-(3-(2,3-Dimethoxybenzyl)thioureido)-N-(2,6-dimethoxy-pyrimidin-4-yl) benzenesulfonamide (**3g**)

This compound was obtained as yellow powder from ethanol; yield 88%; m.p. 151.1 °C. IR: 3292, 3181 (NH), 3047 (arom.), 2986, 2866, 2831 (aliph.), 1587 (CN), 1388, 1172 (SO_2_), 1228 (CS). ^1^H-NMR: δ 3.75, 3.77, 3.80 [3s, 12H, 4OCH_3_], 4.3 [s, 2H, CH_2_],6.8 [s, 1H, H-pyrimidine], 6.9–8.0 [m, 7H, Ar–H], 8.1, 8.3 [2s, 3H, SO_2_NH + 2NH];^13^C-NMR: 40.4, 56.1 (2), 56.7, 60.5, 83.4, 112.3, 120.7 (2), 124.3 (2), 127.0, 128.1 (2), 133.1, 140.2, 146.6, 146.7, 161.4, 165.4, 169.5, 184.6. Anal. Calcd. for C_22_H_25_N_5_O_6_S_2_: C, 50.86%; H, 4.85%; N, 13.48%; S, 12.34%. Found: C, 50.80%; H, 4.80%; N, 13.40%; S, 12.30%.

##### N-(2,6-Dimethoxy-pyrimidin-4-yl)-4-(3-phenethyl-thioureido)benzenesulfonamide (**3h**)

This compound was obtained as yellow powder from ethanol; yield 85%; m.p. 189.7 °C. IR: 3367, 3238 (NH), 3100 (arom.), 2981, 2811 (aliph.), 1635 (CN), 1394, 1130 (SO_2_), 1274 (CS). ^1^H-NMR: δ 2.7, 3.5 [2t, 4H, 2CH_2_, *J* = 8 Hz], 3.63, 3.65 [2s, 6H, 2OCH_3_], 6.6 [s, 1H, H-pyrimidine], 7.1–8.1 [m, 9H, Ar–H], 9.7 [s, 1H, SO_2_NH], 11.0 [s, 2H, 2NH]. ^13^C-NMR: 35.4, 40.4, 55.6, 55.9, 85.0, 124.1 (2), 126.6, 128.8 (2), 129.0 (2), 129.7 (2), 132.7, 140.1, 142.3, 161.5, 165.4, 170.2, 181.8. Anal. Calcd. for C_21_H_23_N_5_O_4_S_2_: C, 53.26%; H, 4.90%; N, 14.79%; S, 13.54%. Found: C, 53.26%; H, 4.90%; N, 14.79%; S, 13.54%.

##### 4-(3-Benzo[1,3]dioxol-5-yl-thioureido)-N-(2,6-dimethoxy-pyrimidin-4-yl)benzenesulfonamide (**3i**)

This compound was obtained as yellow powder from ethanol; yield 85%; m.p. 247.1 °C. IR: 3385, 3169 (NH), 3064 (arom.), 2910, 2895 (aliph.), 1618 (CN), 1381, 1124 (SO_2_), 1240 (CS). ^1^H-NMR: δ 3.82, 3.84 [2s, 6H, 2OCH_3_], 5.9 [s, 1H, H-pyrimidine], 6.0 [s, 2H, O–CH_2_–O], 6.7–8.4 [m, 7H, Ar–H], 9.8 [s, 1H, SO_2_NH], 10.7, 11.4 [2s, 2H, 2NH]. ^13^C-NMR: 55.4, 56.5, 83.7, 101.2, 109.3, 113.6, 118.2, 120.9 (2), 128.8 (2), 129.1, 136.7, 142.6, 146.8, 148.2, 152.9, 167.5, 172.4, 182.0. Anal. Calcd. for C_20_H_19_N_5_O_6_S_2_: C, 49.07%; H, 3.91%; N, 14.31%; S, 13.10%. Found: C, 49.00%; H, 3.90%; N, 14.30%; S, 13.10%.

##### 4-(3-Benzo[1,3]dioxol-4-yl-methyl-thioureido)-N-(2,6-dimethoxy-pyrimidin-4-yl)benzenesulfonamide (**3j**)

This compound was obtained as yellow powder from ethanol; yield 89%; m.p. > 360 °C. IR: 3410, 3371 (NH), 3100 (arom.), 2966, 2889 (aliph.), 1553 (CN), 1376, 1128 (SO_2_), 1251 (CS). ^1^H-NMR: δ 3.61, 3.64 [2s, 6H, 2OCH_3_], 4.2 [s, 2H, CH_2_NH], 6.0 [s, 2H, O–CH_2_–O], 6.6 [s, 1H, H-pyrimidine], 7.0–8.5 [m, 7H, Ar–H], 9.9 [s, 1H, SO_2_NH], 10.3, 12.6 [2s, 2H, 2NH]; ^13^C-NMR: 52.4, 54.3, 54.6, 82.4, 101.3, 108.5, 108.9, 120.6, 124.6 (2), 128.9 (2), 129.7, 133.2, 141.0, 143.9, 146.7, 160.5, 161.7, 163.6, 173.0. Anal. Calcd. for C_21_H_21_N_5_O_6_S_2_: C, 50.09%; H, 4.20%; N, 13.91%; S, 12.73%. Found: C, 50.09%; H, 4.20%; N, 13.90%; S, 12.70%.

##### N-(2,6-Dimethoxy-pyrimidin-4-yl)-4-(3-naphthalen-1-yl-thioureido)benzenesulfonamide (**3k**)

This compound was obtained as yellow powder from ethanol; yield 81%; m.p. 152.0 °C. IR: 3354, 3232 (NH), 3051 (arom.), 2951, 2836 (aliph.), 1620 (CN), 1394, 1184 (SO_2_), 1247 (CS). ^1^H-NMR: δ 3.83, 3.86 [2s, 6H, 2OCH_3_], 5.9 [s, 1H, H-pyrimidine], 6.9–8.4 [m, 11H, Ar–H], 9.8 [s, 1H, SO_2_NH], 11.8 [s, 2H, 2NH]. ^13^C-NMR: 55.3, 56.5, 86.0, 107.9, 115.8, 122.7, 123.2 (2), 124.1, 125.9, 126.3, 127.1, 127.9, 128.2 (2), 130.0, 134.6, 143.1 (2), 163.7, 165.5, 173.6, 181.0. Anal. Calcd. for C_23_H_21_N_5_O_4_S_2_: C, 55.74%; H, 4.27%; N, 14.13%; S, 12.94%. Found: C, 55.70%; H, 4.20%; N, 14.10%; S, 12.90%.

##### N-(2,6-Dimethoxy-pyrimidin-4-yl)-4-(3-(5,6,7,8-tetrahydro-naphthalen-1-yl)thioureido)benzenesulfonamide (**3l**)

This compound was obtained as brown powder from ethanol; yield 83%; m.p. 257.9 °C. IR: 3400, 3309 (NH), 3082 (arom.), 2931, 2835 (aliph.), 1616 (CN), 1377, 1136 (SO_2_), 1280 (CS). ^1^H-NMR: δ 1.6–2.8 [m, 8H, 4CH_2_ cyclo], 3.81, 3.84 [2s, 6H, 2OCH_3_], 6.2 [s, 1H, H-pyrimidine], 6.6-8.0 [m, 7H, Ar–H], 8.8 [s, 1H, SO_2_NH], 11.7, 11.9 [2s, 2H, 2NH]. ^13^C-NMR: 22.6 (2), 23.3, 29.8, 55.1, 55.6, 82.7, 117.4, 119.5 (2), 120.8, 124.0, 127.4 (2), 137.1, 137.5, 137.6 (2), 146.5, 161.1, 161.8, 170.9, 179.8. Anal. Calcd. for C_23_H_25_N_5_O_4_S_2_: C, 55.29%; H, 5.04%; N, 14.02%; S, 12.84%. Found: C, 55.20%; H, 5.00%; N, 14.00%; S, 12.80%.

##### N-(2,6-Dimethoxy-pyrimidin-4-yl)-4-(3-indan-5-yl-thioureido)benzenesulfonamide (**3m**)

This compound was obtained as yellow powder from ethanol; yield 80%; m.p. 119.1 °C. IR: 3458, 3253 (NH), 3061 (arom.), 2945, 2889, 2839 (aliph.), 1627 (CN), 1396, 1130 (SO_2_), 1273 (CS). ^1^H-NMR: δ 1.9–2.0 [m, 2H, CH_2_ cyclo], 2.7–2.8 [m, 4H, 2CH_2_ cyclo], 3.79, 3.80 [2s, 6H, 2OCH_3_], 5.8 [s, 1H, H-pyrimidine], 6.6–8.4 [m, 7H, Ar–H], 9.9 [s, 1H, SO_2_NH], 11.4, 12.3 [2s, 2H, 2NH]. ^13^C-NMR: 25.6, 32.2 (2), 55.4, 56.5, 79.8, 116.8 (2), 124.6 (2), 124.9, 128.3 (2), 137.2, 138.4, 144.6 (2), 145.7, 154.8, 170.0, 172.4, 183.0. Anal. Calcd. for C_22_H_23_N_5_O_4_S_2_: C, 54.42%; H, 4.77%; N, 14.42%; S, 13.21%. Found: C, 54.40%; H, 4.70%; N, 14.40%; S, 13.20%.

##### N-(2,6-Dimethoxy-pyrimidin-4-yl)-4-(3-(2-(1-methyl-1H-pyrrol-2-yl)ethyl)thioureido)benzenesulfonamide (**3n**)

This compound was obtained as brown powder from ethanol; yield 86%; m.p. > 360 °C. IR: 3410, 3216 (NH), 3100 (arom.), 2943, 2839 (aliph.), 1595 (CN), 1386, 1126 (SO_2_), 1262 (CS). ^1^H-NMR: δ 2.8 [t, 2H, CH_2_, *J* = 8 Hz], 3.5 [s, 3H, N–CH_3_], 3.70, 3.72 [2s, 6H, 2OCH_3_], 3.9 [t, 2H, CH_2_–NH, *J* = 8 Hz], 5.9–6.7 [m, 3H, 3H-pyrrole], 6.8 [s, 1H, H-pyrimidine], 7.0–8.2 [m, 4H, Ar–H], 9.9 [s, 1H, SO_2_NH], 11.2, 12.3 [2s, 2H, 2NH]. ^13^C-NMR: 25.4, 35.6, 44.2, 52.9, 53.8, 83.6, 105.1, 107.3, 123.8, 123.9 (2), 125.5, 128.0 (2), 134.2, 143.1, 162.6, 163.9, 173.4, 182.3. Anal. Calcd. for C_20_H_24_N_6_O_4_S_2_: C, 50.41%; H, 5.08%; N, 17.63%; S, 13.46% Found: C, 50.40%; H, 5.00%; N, 17.60%; S, 13.46%.

##### N-(2,6-Dimethoxy-pyrimidin-4-yl)-4-(3-(2-pyrrolidin-1-yl-ethyl)thioureido) benzenesulfonamide (**3o**)

This compound was obtained as brown powder from ethanol; yield 86%; m.p. 280.0 °C. IR: 3342, 3323 (NH), 3045 (arom.), 2972, 2856 (aliph.), 1622 (CN), 1386, 1180 (SO_2_), 1274 (CS). ^1^H-NMR (DMSO-*d*_*6*_): δ 1.6–1.8 [m, 4H, CH_2_–CH_2_–pyrrolidine], 2.51–2.56 [m, 4H, CH_2_–N–CH_2_pyrrolidine], 2.62–2.68 [m, 2H, N–CH_2_], 3.4 [t, 2H, CH_2_–NH, *J* = 8 Hz], 3.82, 3.85 [2s, 6H, 2OCH_3_], 6.5 [s, 1H, H-pyrimidine], 6.9–7.9 [m, 4H, Ar–H], 9.8 [s, 1H, SO_2_NH], 11.4 [s, 2H, 2NH]; ^13^C-NMR: 23.3 (2), 40.4, 51.6, 52.9 (2), 53.5, 54.1, 81.7, 120.7 (2), 127.7 (2), 136.4, 140.2, 161.0, 166.3, 170.6, 180.5. Anal. Calcd. for C_19_H_26_N_6_O_4_S_2_: C, 48.91%; H, 5.62%; N, 18.01%; S, 13.74%. Found: C, 48.90%; H, 5.60%; N, 18.00%; S, 13.70%.

##### N-(2,6-Dimethoxy-pyrimidin-4-yl)-4-(3-(9,10-dioxo-9,10-dihydro-anthracen-2-yl)thioureido)benzenesulfonamide (**3p**)

This compound was obtained as yellow powder from ethanol; yield 84%; m.p. 299.6 °C. IR: 3433, 3348, 3219 (NH), 3064 (arom.), 2921, 2871 (aliph.), 1705, 1672 (2CO), 1625 (CN), 1338, 1178 (SO_2_), 1280 (CS). ^1^H-NMR: δ 3.70, 3.73 [2s, 6H, 2OCH_3_], 6.6 [s, 1H, H-pyrimidine], 6.9–8.4 [m, 11H, Ar–H], 10.8 [s, 1H, SO_2_NH], 11.6, 11.9 [2s, 2H, 2NH]. ^13^C-NMR: 57.2, 57.8, 83.7, 118.5, 121.6 (2), 126.7 (2), 130.0, 133.4 (2), 133.7 (2), 134.1 (2), 134.7 (2), 135.3 (2), 141.0 (2), 155.1, 162.7, 172.4, 180.5, 183.7 (2). Anal. Calcd. for C_27_H_21_N_5_O_6_S_2_: C, 56.34%; H, 3.68%; N, 12.17%; S, 11.14%. Found: C, 56.30%; H, 3.60%; N, 12.10%; S, 11.10%.

##### N-(2,6-Dimethoxy-pyrimidin-4-yl)-4-(3-(2-methyl-1,3-dioxo-2,3-dihydro-1H-isoindol-5-yl)thioureido)benzenesulfonamide (**3q**)

This compound was obtained as yellow powder from ethanol; yield 85%; m.p. 194.7 °C. IR: 3475, 3363, 3219 (NH), 3055 (arom.), 2951, 2809 (aliph.), 1755, 1689 (2CO), 1622 (CN), 1382, 1157 (SO_2_), 1271 (CS). ^1^H-NMR: δ 3.0 [s, 3H, N-CH_3_], 3.70, 3.72 [2s, 6H, 2OCH_3_], 6.4 [s, 1H, H-pyrimidine], 6.8- 8.0 [m, 7H, Ar–H], 9.0 [s, 1H, SO_2_NH], 10.9 [s, 2H, 2NH]; ^13^C-NMR: 23.7, 55.4, 55.6, 81.2, 117.3, 125.0 (2), 127.9 (2), 128.0 (2), 130.7, 135.0 (2), 142.1 (2), 162.7, 168.5 (2), 168.8, 174.0, 185.2. Anal. Calcd. for C_22_H_20_N_6_O_6_S_2_: Anal. Calcd. for C_27_H_21_N_5_O_6_S_2_ C, 49.99%; H, 3.81%; N, 15.90%; S, 12.13%. Found: C, 49.90%; H, 3.80%; N, 15.90%; S, 12.10%.

##### N-(2,6-Dimethoxy-pyrimidin-4-yl)-4-(3-(3,5-dipyridin-2-yl- [1,2,4]triazol-4-yl)thioureido)benzenesulfonamide (**3r**)

This compound was obtained as brown powder from ethanol; yield 83%; m.p. 286.0 °C. IR: 3411, 3188 (NH), 3100 (arom.), 2960, 2829 (aliph.), 1622 (CN), 1377, 1138 (SO_2_), 1249 (CS). ^1^H-NMR: δ 3.70, 3.74 [2s, 6H, 2OCH_3_], 6.6 [s, 1H, H-pyrimidine], 7.1–8.6 [m, 12H, Ar–H], 9.8 [s, 1H, SO_2_NH], 10.9, 11.9 [s, 2H, 2NH]. ^13^C-NMR: 56.7, 56.9, 85.6, 121.9 (2), 125.0 (2), 126.1 (2), 128.8 (2), 137.9, 138.2 (2), 140.6, 143.1 (2), 150.0 (2), 151.6 (2), 162.8, 164.0, 174.3, 187.1. Anal. Calcd. for C_25_H_22_N_10_O_4_S_2_: Anal. Calcd. for C_27_H_21_N_5_O_6_S_2_ C, 50.84%; H, 3.75%; N, 23.71%; S, 10.86%. Found: C, 50.80%; H, 3.7 0%; N, 23.70%; S, 10.86%.

##### N-(2,6-Dimethoxy-pyrimidin-4-yl)-4-(3-(4-morpholin-4-yl-phenyl)thioureido)benzenesulfonamide (**3s**)

This compound was obtained as brown powder from ethanol; yield 81%; m.p. 184.0 °C. IR: 3377, 3304 (NH), 3069 (arom.), 2962, 2852 (aliph.), 1635 (CN), 1379, 1151 (SO_2_), 1263 (CS). ^1^H-NMR: δ 3.1–3.2 [m, 4H, CH_2_–N–CH_2_], 3.60–3.68 [m, 4H, CH_2_–O–CH_2_], 3.79, 3.85 [2s, 6H, 2OCH_3_], 5.9 [s, 1H, H-pyrimidine], 6.5–8.3 [m, 8H, Ar–H], 9.9 [s, 1H, SO_2_NH], 10.6, 11.0 [2s, 2H, 2NH]. ^13^C-NMR: 49.7 (2), 55.4, 55.6, 66.6 (2), 84.0, 112.9 (2), 119.8 (2), 124.9 (2), 127.6, 129.8 (2), 133.7, 140.0, 146.8, 155.9, 167.0, 173.4, 179.7. Anal. Calcd. for C_23_H_26_N_6_O_5_S_2_: C, 52.06%; H, 4.94%; N, 15.84%; S, 12.09%. Found: C, 52.00%; H, 4.90%; N, 15.80%; S, 12.00%.

##### 4-(3-Adamantan-1-yl-thioureido)-N-(2,6-dimethoxy-pyrimidin-4-yl) benzenesulfonamide (**3t**)

This compound was obtained as white crystals from ethanol; yield 81%; m.p. 164.6 °C. IR: 3346, 3176 (NH), 3100 (arom.), 2910, 2852 (aliph.), 1625 (CN), 1375, 1186 (SO_2_), 1236 (CS). ^1^H-NMR: δ 1.6–1.7 [m, 3H, 3CH-adamantyl], 1.8-2.1 [m, 12H, 6CH_2_-adamantyl], 3.80, 3.83 [2s, 6H, 2OCH_3_], 5.9 [s, 1H, H-pyrimidine], 7.1–8.4 [m, 4H, Ar–H], 9.8 [s, 1H, SO_2_NH], 11.4, 12.6 [2s, 2H, 2NH]. ^13^C-NMR: 29.4 (3), 35.2 (3), 42.5 (3), 43.4, 59.2, 63.4, 85.3, 120.6 (2), 130.7 (2), 136.8, 142.1, 153.9, 169.5, 172.8, 182.1. Anal. Calcd. for C_23_H_29_N_5_O_4_S_2_: C, 54.85%; H, 5.80%; N, 13.91%; S, 12.73%. Found: C, 54.80%; H, 5.80%; N, 13.90%; S, 12.70%.

### Antimycobacterial activity

The *M. tuberculosis* (RCMB 010126) strain was provided from the culture collection of the Regional Center for Mycology and Biotechnology (RCMB), Al-Azhar University (Cairo, Egypt). The antimycobacterial activity of the synthesized compounds was performed using the microplate Alamar blue assay technique [35] with minor modifications which were performed in sterile 96 well microplates. To prevent dehydration in experimental wells, the outer perimeter wells of the plate were filled with sterile water. 100 µL of 10^5^ CFU/mL *M. tuberculosis* inoculum was added to the wells. For detecting the antimycobacterial activity of the synthesized compounds, 100 µL (at 200 µg/mL) dissolved in dimethyl sulfoxide was then added to the wells. Isoniazid was used as a positive control. Also, additional control wells consisted of bacteria only was performed. Five replicates were tested for each treatment along with the controls. The plates were then incubated for at least 4 days at 37 °C. After the end of incubation period, 20 µL of Alamar blue solution (Alamar Biosciences/Accumed, Westlake, OH, USA) and 12.5 µL of 20% Tween 80 (Merck, Darmstadt, Germany) were added to all the wells of the plate. The plates were then incubated again at 37 °C for 24 h in the dark. The results were recorded at 24 h post-reagent addition at 590 nm. The percent of inhibition was defined as: $1 - \left( {{\text{mean of the test well}}/{\text{mean of B wells}}} \right) \; \times 100$. For the determination of the minimum inhibitory concentrations, stock solutions of the tested compounds were prepared in dimethyl sulfoxide and subsequent twofold dilutions were performed in the 96 well microplates to achieve concentrations ranged from 200 to 0.1 µg/mL. These concentrations were tested for their activity with estimation of the inhibition % as described before. The lowest drug concentration causing inhibition of *M. tuberculosis* was considered as the MIC.

### Molecular docking

The molecular model of the new thiourea derivatives was done using MOE software suite 10.2008. Following geometry optimization, a systematic conformational search was carried out to RMS gradient of 0.05 Å with energy minimization of the resultant conformations employing the ConfSearch module implemented in MOE. All molecular mechanics computations were performed with the Merck Force Field (MMFF94s). The crystallographic structure of *M. tuberculosis* enoyl reductase InhA in complex with *N*-{1-[(2-chloro-6-fluorophenyl)methyl]-*1H*-pyrazol-3-yl]-5-[(1S)-1-(3-methyl-*1H*-pyrazol-1-yl)ethyl]-1,3,4-thiadiazol-2-amine (GSK 625) was obtained from the Protein Data Bank (PDB ID: **5JFO**). Water molecules were ignored and hydrogen atoms were added to the enzyme and partial charges were calculated. Validation followed by docking of the compounds into the active site were carried out, after removing the co-crystallized ligand. The target protein was kept rigid, while the ligands adopt 50 separate docking simulations using default parameters. The conformations were chosen based on their S score, and appropriate fitting with the relevant amino acids in the binding pocket.

## Conclusion

A new series of *N*-(2,6-dimethoxypyrimidin-4-yl)-4-(3-(aryl)thioureido) benzenesulfonamides **3a**–**t** was synthesized. The target compounds were designed and synthesized as potential antitubercular agents. Compounds **3i** and **3s** were found to be the most potent in this study, the reference drug used in this study was isoniazid. Compound **3i** (MIC = 3.13 µg/mL), was the most potent followed by compound **3s** (MIC = 6.25 µg/mL). Also, the docking study showed that all the docked compounds exhibited similar binding interaction as those previously reported by the co-crystallized ligand when docked into the active site of *M. tuberculosis* enoyl reductase InhA.
